# Geometry‐Encoded Programmable Mechanics in Single‐Material Dual‐Phase Metamaterials

**DOI:** 10.1002/smll.74687

**Published:** 2026-07-24

**Authors:** Miao Zhao, Na Qiu, Xuhui Zhang, Bei Peng, Zhi Zeng, Xinwei Li, Qing Li

**Affiliations:** ^1^ School of Mechanical and Electrical Engineering University of Electronic Science and Technology of China Sichuan China; ^2^ School of Aerospace Mechanical and Mechatronic Engineering The University of Sydney Sydney New South Wales Australia; ^3^ Newcastle University in Singapore Faculty of Science, Agriculture, and Engineering Newcastle University Newcastle upon Tyne UK

**Keywords:** additive manufacturing, inverse design, machine learning, metamaterial, programmable mechanical property

## Abstract

Programmable metamaterials that exhibit prescribed mechanical responses and adaptive deformation under external loading are highly desirable for multifunctional engineering applications. However, most existing designs rely on multi‐material systems, which pose significant fabrication challenges with conventional additive manufacturing. Inspired by the unique soft‐hard heterogeneous architecture of nacre, this study introduces a novel class of dual‐phase (DP) metamaterials where spatially encoded soft and hard phases are realized through bending‐dominated and stretching‐dominated lattice architectures, respectively. By systematically varying the spatial coding patterns of soft‐hard phases, representative DP metamaterials are shown to exhibit programmable nonlinear mechanical responses and tailored failure processes, achieved through geometry‐based mechanical encoding governed by phase interactions and internal stress redistribution. Notably, the engineered sequenced failure processes and phase‐coupling‐induced strengthening effects lead to significantly enhanced energy absorption compared with the constituent architectures, while enabling customizable plateau stress. To efficiently explore the vast design space of DP metamaterials, a data‐driven framework is then developed to model the relationship between spatial encodings and nonlinear mechanical responses. The trained model enables rapid and accurate inverse design of DP metamaterials matching the complex target responses for multifunctional applications. Overall, this work establishes a new geometry‐based strategy for achieving highly programmable mechanical responses in single‐material metamaterials.

## Graphical abstract

Inspired by the unique soft‐hard heterogeneous architecture of nacre, this study proposes a novel class of dual‐phase (DP) metamaterials. The DP metamaterials exhibit programmable nonlinear mechanical responses, tailored failure processes, and enhanced energy absorption by systematically varying the spatial coding patterns of soft‐hard unit cells.

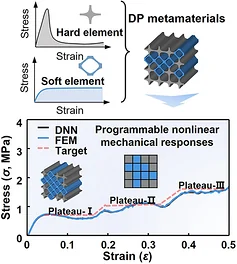

## 1 Introduction

Lattice metamaterials exhibit a wide range of multifunctional properties enabled by their distinctive and highly tailorable internal geometries, which are difficult or even impossible to achieve by intrinsic materials alone. These properties include ultra‐lightweight characteristics [1, 2], exceptional stretchability [3, 4], high impact resistance [5, 6], shape memory behavior [7, 8], and negative Poisson's ratios [9, 10]. The advent of additive manufacturing (AM) technologies has enabled the direct and precise fabrication of lattice metamaterials with complex, controllable geometries in a “bottom‐up” manner, thereby accelerating their adoption in engineering applications such as aerospace, automotive, mechanical, and biomedical fields.

To date, a broad spectrum of lattice metamaterials, including strut‐based [11, 12, 13], shell‐based [14, 15], and plate‐based [16, 17], has been proposed and analyzed. Fundamental studies have demonstrated that the mechanical responses of lattice metamaterials are predominantly governed by their architectural design. Based on the dominant deformation mechanism of the internal geometries under external load, the lattice metamaterials can be broadly classified as stretching‐dominated and bending‐dominated. Bending‐dominated lattices typically tend to exhibit high compression toughness but relatively low stiffness and strength, making them better suited for energy absorption applications [18]. In contrast, stretching‐dominated lattices offer high stiffness and strength, but often suffer from abrupt post‐yield failure and catastrophic structural collapse under compression, which limits their practical use primarily to lightweight load‐bearing structures [19]. To simultaneously improve strength and toughness of lattice metamaterials, various design strategies have been explored, including the introduction of fillets [20], hybridization of different unit‐cell types [21, 22], and optimization of strut cross‐sections [23, 24]. Nevertheless, these conventional approaches provide limited tunability of mechanical characteristics and are often fail to meet the diverse and increasingly demanding requirements of advanced engineering applications.

**FIGURE 1:**
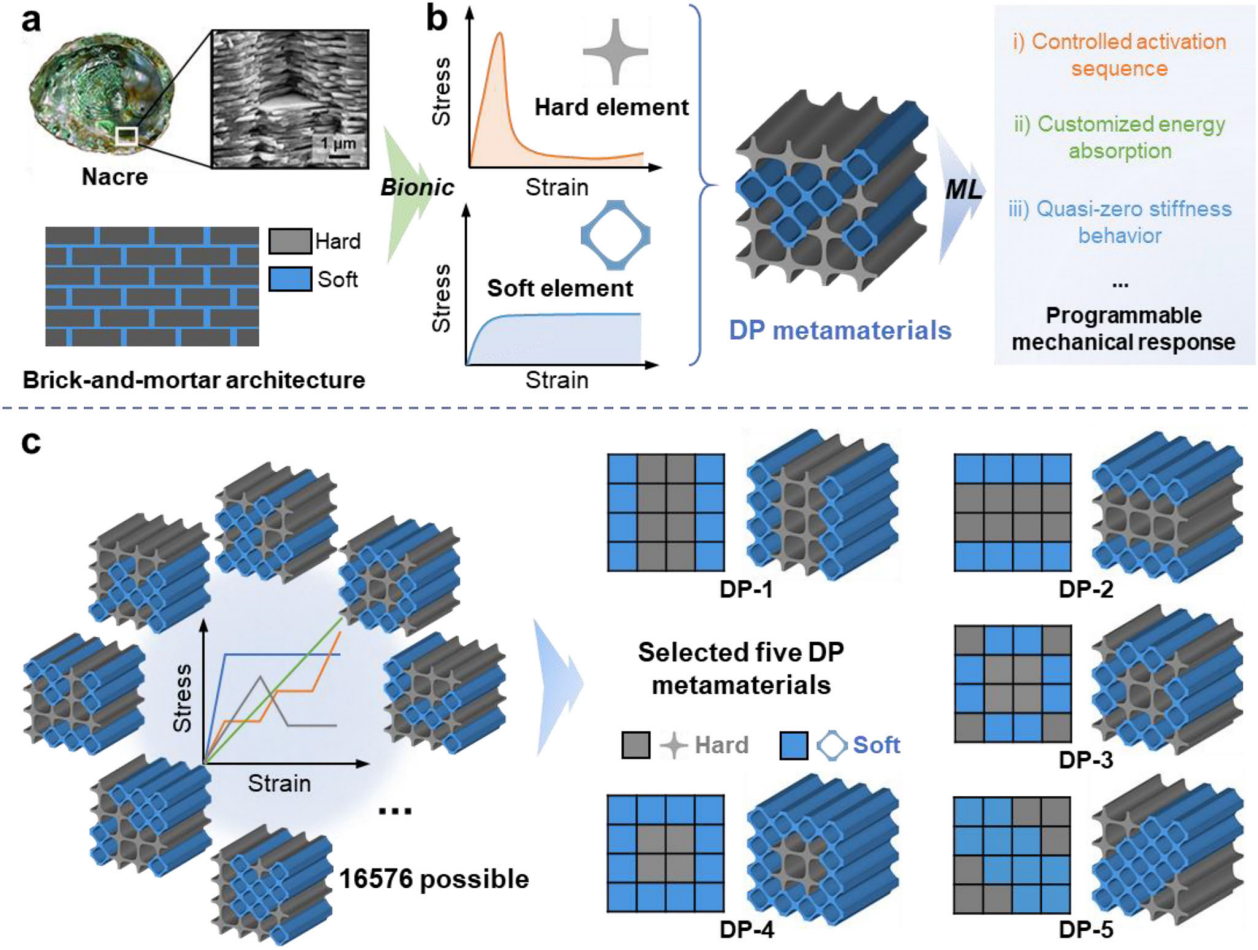
Design concept of nacre‐inspired dual‐phase (DP) metamaterials. (a) Schematic of the hierarchical nacre structure composed of hard aragonite plates and soft protein layers [34]. (b) Illustration of the proposed DP metamaterials comprising soft (shell‐based lattices) and hard (strut‐based lattices) elements. (c) Representative DP metamaterials with varied spatial coding patterns, and five designs are selected for detailed investigation.

Natural materials routinely achieve exceptional combinations of mechanical properties that far surpass those of many engineering materials available today [25, 26]. Inspired by natural and biological systems, numerous bioinspired design concepts for lattice metamaterials have been proposed [27, 28, 29]. Among them, nacre is widely recognized as one of the toughest natural armor materials, consisting of soft biopolymers and hard mineral platelets arranged in a characteristic “brick‐and‐mortar” architecture [30, 31], as shown in Figure 1a. This hierarchical structure enables to attain outstanding strength and damage tolerance [32], where the hard and stiff mineral platelets primarily bear load while the soft biopolymer layers dissipate energy and inhibit crack propagation. Motivated by these mechanisms, increasing attention has been devoted to the development of dual‐phase (DP) metamaterials, in which two distinct materials phases are topologically interconnected within the design domain, such as ceramic‐polymer [33], metal‐metal [34], metal‐polymer [35], and polymer‐polymer [36, 37, 38] systems. These interconnected configurations not only promote efficient stress transfer and damage delocalization, leading to enhanced strength and ductility beyond that achievable by either constituent phase alone. Moreover, by tailoring the distribution and interaction of the constituent phase, DP metamaterials can exhibit programmable mechanical responses to satisfy diverse functional requirements of engineering applications, capabilities that are difficult to realize with traditional single‐material metamaterials. Nevertheless, most existing DP metamaterials rely on to multi‐material fabrication rather than explicitly architected topologies, which presents significant challenges for direct fabrication using a single material AM process.

To overcome the current limitations of existing DP metamaterial design, this study proposes a novel class of soft‐hard DP metamaterials made of a single material, inspired by the unique “brick‐and‐mortar” architecture of nacre. In this concept, the effective soft and hard mechanical behaviors are realized by integrating bending‐dominated and stretching‐dominated unit cells, respectively, within a unified lattice architecture. The programmable mechanical responses are achieved through deliberate design of the soft‐hard unit‐cells and their spatial arrangements, enabling broad compatibility with a wide range of AM processes. It should be noted that, while previous nacre‐inspired studies have demonstrated innovative strategies of achieving high strength and post‐yield stability, the strengthening mechanisms and coupled deformation behaviors arising from interactions between soft and hard units in DP metamaterials remain largely unexplored. Moreover, although structure‐property relationships provide the foundation for encoding desired mechanical responses, the rapidly expanding number of possible coding patterns renders conventional intuition‐based design approaches impractical, thereby fundamentally limiting their applicability in engineering design.

In this work, we propose a systematic design and evaluation of nacre‐inspired DP metamaterials with varied spatial distributions of soft and hard unit‐cells. Geometry‐based encoding of soft‐hard unit‐cells introduces a new paradigm for mechanical programmability, enabling precise control over nonlinear mechanical behavior, energy absorption, and failure process of metamaterials. Notably, the engineered sequenced failure processes and phase‐coupling‐induced enhancement effects lead to significantly improved energy absorption, even exceed that of both purely soft and hard metamaterials of identical volume fraction. Finally, an inverse coding design framework is developed to enable rapid and efficient programming of target mechanical responses in DP metamaterials for diverse engineering applications. Overall, this work not only establishes an effective strategy for unlocking programmable mechanical responses in single‐material metamaterials but also advances fundamental understanding of the deformation and failure mechanisms underlying the nacre's unique “brick‐and‐mortar” architecture.

## 2 Design Principles

The proposed conceptual framework, referred to as a “soft‐hard dual‐phase (DP) metamaterials”, consists of two types of 2D triply periodic minimal surface (TPMS)‐based lattice structures (Figure 1b). The strut‑based primitive lattices function as the “hard” phase, while the shell‐based lattices serve as the “soft” phase. The strut‐based primitive lattices exhibited high stiffness/strength but limited energy absorption capacity, characteristic of a stretching‐dominated architecture. The limited energy absorption capacity of strut‐based primitive lattices was due to a pronounced stress softening during the plateau stage due to local buckling of the struts. Similar phenomenon was also observed in some stretching‐dominated lattices [39]. In contrast, the shell‐based primitive lattices offer a pronounced stress plateau with enhanced energy absorption but relatively low stiffness/strength, typical bending‐dominated lattices. Therefore, by spatially distributing these soft and hard lattice phases within a single structure, the mechanical properties of the DP metamaterials can be effectively encoded through geometry alone.

The 3D models of DP metamaterials were generated using the implicit function representations, with continuous transitions between heterogeneous unit‐cells achieved by a sigmoid function (see the Supporting Information, Section S1). As an illustration, five representative DP metamaterials with the distinct spatial coding patterns of soft and hard elements are presented in Figure 1c. These five designs of DP metamaterials are subsequently used to demonstrate their unique and programmable mechanical responses and deformation behaviors of the proposed DP metamaterials in the following section.

## 3 Results and Discussion

### 3.1 Programmable Nonlinear Mechanical Responses

**FIGURE 2:**
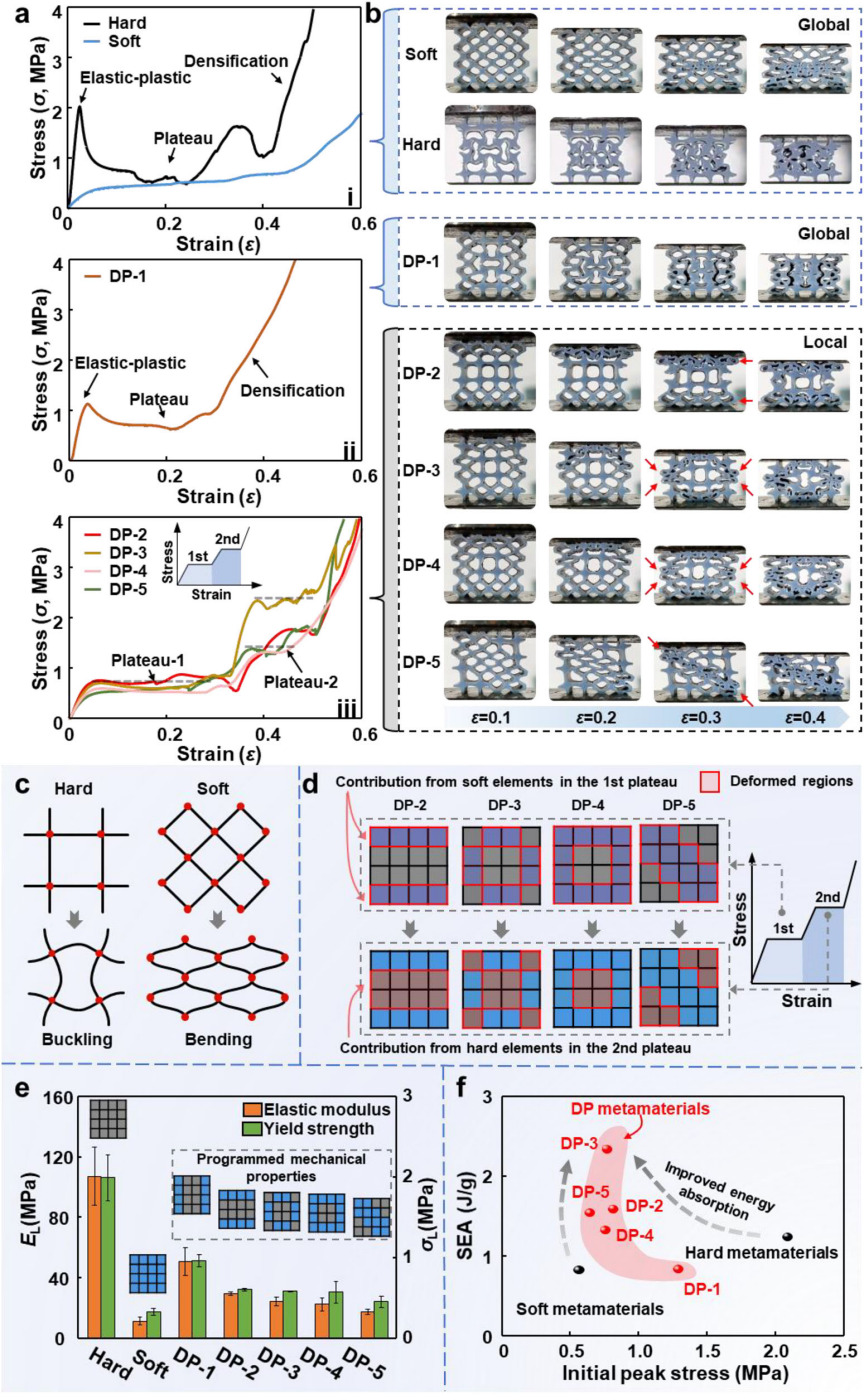
Compressive responses of single‐phase (SP) and DP metamaterials. (a) Stress–strain curves of i) SP and ii, iii) DP metamaterials. (b) Representative deformation modes of SP and DP metamaterials at selected strain values. Schematic illustrations of deformation mechanisms in (c) SP and (d) DP metamaterials. (e) Elastic modulus and yield strength of SP and DP metamaterials. (f) Specific energy absorption (SEA) versus initial peak stress for SP and DP metamaterials.

The experimental investigations were first carried on the single‐phase (SP) metamaterial samples for benchmarks, followed by tests on the five DP configurations as shown in Figure 1c to demonstrate the feasibility of achieving programmable nonlinear responses through dual‐phase variations. Figure 2a,b present the compressive stress‐strain responses and the corresponding deformation modes of the SP and DP metamaterials, respectively. In contrast to the fixed mechanical responses of SP metamaterials, the DP metamaterials exhibited distinct nonlinear mechanical responses that depended strongly on their spatial coding patterns, thereby confirming the successful realization of programmable mechanical responses through controlled spatial arrangements of soft and hard phases.

Under compressive loading, the stress‐strain responses of all the metamaterials can be broadly divided into three regimes: (i) elastic‐plastic deformation, (ii) plateau region, and (iii) densification. For conventional SP metamaterials, the mechanical responses are fully determined by the predefined topological configuration, as illustrated in Figure 2a (i). The hard SP metamaterials exhibited a pronounced stress softening during the plateau stage due to local buckling of the struts (Figure 2c), which is a typical mechanical characteristic of the stretching‑dominated metamaterials. In contrast, the soft metamaterials deformed predominantly through uniform bending of the struts (Figure 2c), resulting in a long and stable stress plateau. Notably, the hard metamaterials underwent inward contraction driven by twist‑like deformation of locally buckled struts, leading to a negative Poisson's effect during compression. Conversely, the soft metamaterials expanded laterally as the struts bent outward.

Although the DP metamaterials are composed of the same soft and hard elements, they displayed markedly different and programmable mechanical responses as a direct consequence of their deliberate spatial arrangement of soft and hard phases, as shown in Figure 2a (ii, iii). For instance, the vertical alignment of hard elements in DP‐1 promoted early buckling, resulting in a rapid loss of load‐bearing capacity during the plateau stage. In contrast, the other four DP configurations maintained a relatively stable plateau stress following initial failure. This behavior arises from the initially localized collapse within the soft phases, where the local bending‑dominated deformation mitigated abrupt reductions in load‐bearing capacity.

Noteworthy, these DP metamaterials exhibited a distinct second plateau region in their stress–strain curves. After the soft phases collapsed, the deformation progressively transformed to the hard phases, thereby enhancing the overall load‐bearing capability, as shown in Figure 2d. This effect is particularly pronounced in the DP‑3 metamaterials, in which the second plateau stress was approximately twice that of the first. Two primary factors contribute to this enhancement. First, the DP‑3 metamaterials contain the highest proportion of hard elements which influence the level of second plateau stress. Second, the hard elements at the corners undergo inward contraction during compression, while the internal hard regions are laterally confined by the surrounding soft elements. This synergistic interactive deformation promotes more effective load transfer and resistance, resulting in a substantial increase in the second plateau stress.

The mechanical properties of the various metamaterials are summarized at Figure 2e. As expected, the hard metamaterials exhibited the highest stiffness and strength, whereas the soft metamaterials displayed the lowest. Specifically, the elastic modulus and yield strength of the hard metamaterials were 9.66 and 6.15 times higher than those of the soft counterparts, respectively. For DP metamaterials, the programmable mechanical properties were achieved through the geometric encoding of soft and hard elements within the DP metamaterials. Notably, the mechanical properties of DP metamaterials are governed not only by the proportion of each phase but also by their spatial arrangement. For example, although DP‐1, DP‐2, and DP‐3 configurations contain the same number of hard elements, their mechanical properties differ significantly. Among them, the DP‐1 metamaterials had the highest elastic modulus and yield strength, which were 111.16% and 66.34% higher than those of DP‐3 metamaterials, respectively. These results highlight the critical role of spatial coding patterns in tuning mechanical properties, and the topological arrangement of soft and hard elements dictated the load‐transfer pathways under the compressive load, which will be further discussed in the next section.

For energy absorption, the initial peak stress and specific energy absorption (SEA) are two key indicators commonly used to evaluate the performances of metamaterials. The SEA is generally defined as:

(1) $$SEA=\frac{\int_{0}^{\varepsilon_{D}}\sigma \left(\varepsilon \right)}{\rho }\;$$

where ε_*D*_ denotes the densification strain, and ρ is the density of the metamaterials. Figure 2f summarizes the energy absorption characteristics of both the SP and DP metamaterials. Apparently, the hard metamaterials exhibited the highest initial peak stress but a relatively low *SEA*, which was unfavorable for energy absorption applications. An excessively high initial peak stress may cause premature structural failure or damage prior to the onset of a stable plateau regime. The low energy absorption capability is primarily attributed to their stretching‐dominated deformation behaviors and negative Poisson's effect. Under compression, the vertical struts were prone to buckling, resulting in a rapid reduction of load‐bearing capacity following the initial failure. Furthermore, the negative Poisson effect induced the pronounced lateral contraction, leading to an early densification strain and further reduced SEA. In contrast, the soft metamaterials exhibited the lowest initial peak stress due to the progressive bending‐dominated deformation of the struts, but their SEA remained lowest as well.

For the DP metamaterials, the programmable energy absorption was successfully achieved through deliberate tailoring the spatial arrangement of soft and hard elements. Among the five configurations investigated, the DP‐3 metamaterials exhibited the highest SEA, outperforming both soft and hard SP metamaterials. Specifically, the SEA of DP‐3 metamaterials was enhanced by 88.54% and 180.9% compared to the soft and hard SP metamaterials, respectively. Moreover, the initial peak stress of DP‐3 metamaterials was significantly lower than that of the hard metamaterials, with a reduction of 62.85% which is highly desirable for impact and energy‐absorption applications.

The superior energy absorption performance of the DP‐3 metamaterials originates from their unique multi‐stage deformation mechanisms. During the initial loading stage, the soft phases preferentially collapsed effectively reducing initial peak force, while the hard phases underwent lateral deformation that progressively enhanced the load‐bearing capacity. This sequentially programmed deformation process of soft and hard phases significantly increased the energy absorbed, resulting in a pronounced coupling enhancement effect, often described “1+1>2”. A similar soft–hard synergistic deformation mechanism has been also observed in natural nacre, where the hierarchical arrangement of compliant (soft) and stiff (hard) constituents significantly enhances the impact resistance [31].

Indeed, the energy absorption performance of DP metamaterials is highly sensitive to the spatial coding patterns of the soft and hard phases. Despite containing the same number of hard elements, the DP‐1 metamaterials exhibited inferior energy absorption behavior, characterized by a higher initial peak stress but lower SEA than DP‐3. These results confirmed that a rational design and strategic topological arrangement of soft and hard elements are essential for fully exploiting coupled deformation mechanisms, thereby achieving substantial enhancements in the energy absorption capability of DP metamaterials.

### 3.2 Programmed Failure Process

**FIGURE 3:**
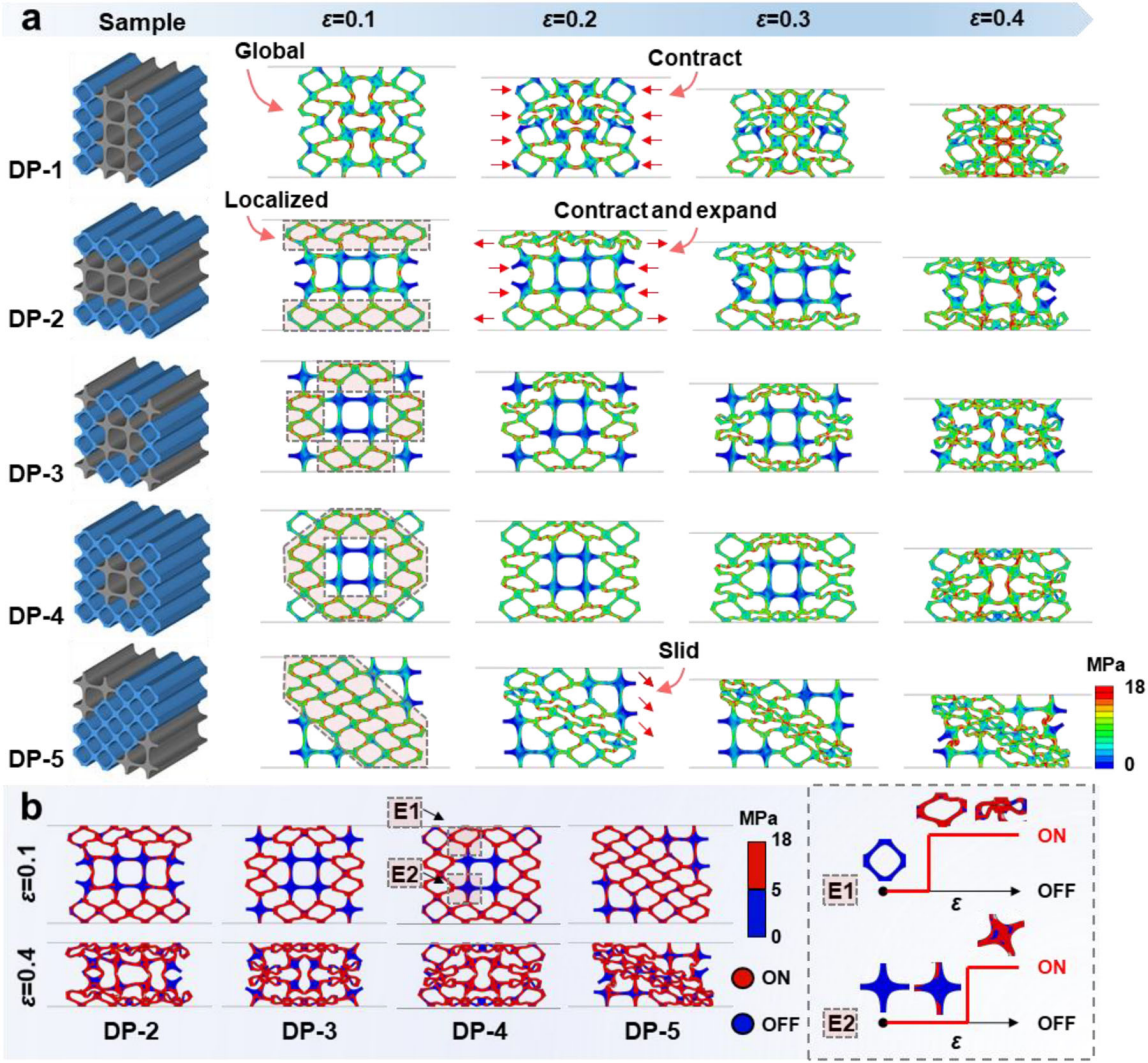
Programmable failure process of DP metamaterials. (a) The finite element predicted stress distribution of DP metamaterials during the compression process. (b) Stress distribution at a prescribed stress threshold, illustrating the sequential activation and failure mechanisms of DP metamaterials at different strain levels.

Here, we elucidate the mechanisms influencing the spatial coding on the nonlinear mechanical responses of the metamaterials. To this end, stress distributions under compressive loading were obtained using the finite element method (FEM), as shown in Figure 3a. Owing to the distinct spatial arrangement of soft and hard elements, the DP metamaterials exhibited markedly different deformation behaviors and failure processes.

For the DP‐1 metamaterials, a largely homogeneous deformation mode was observed at a at a compressive strain of 0.1, with stress distributed relatively uniformly across both soft and hard elements. This behavior is attributed to the uniform vertical alignment of the two phases along the loading direction, which enables direct transmission of compressive forces between the elements of same phase. Consequently, the soft and hard elements deformed simultaneously rather than sequentially. With further compression, the internal hard elements exhibited a pronounced negative Poisson's effect, leading to rapid lateral contraction and early overall compaction of the metamaterials. These observations confirm previous findings that the DP‐1 metamaterials entered the densification stage at a relatively low strain due to its uniform phase arrangement.

In contrast, the other four types of DP metamaterial configurations exhibited localized deformation within the soft phases at a compressive strain of 0.1. With increasing strain, the failures progressively propagated from the soft phases to the hard phases, giving rise to a sequenced failure process confined to local regions. This behavior closely resembles the deformation and failure characteristics observed in conventional DP metallic alloys, such as DP steels [40]. Moreover, the topological arrangement of soft and hard phases plays a decisive role in determining the localized deformation patterns. For instance, the DP‐2 metamaterials exhibited lateral expansion at the top and bottom layers, while the middle layers contracted, producing a localized negative Poisson's ratio. Such spatially heterogeneous mechanical responses are unprecedented in natural materials and highlight the capabilities of architected metamaterials to not only emulate but also surpass biological design principles.

In the DP‐3 and DP‐4 metamaterials, the outer soft phases fully collapsed under compression, whereas the internal hard regions experienced minimal deformation. This sandwich‐like architecture effectively shielded the interior structure from damage during energy absorption, demonstrating the protective function enabled by soft‐hard phase coding for resisting localized damage. Interestingly, despite being fabricated from a tough and ductile resin, the DP‐5 metamaterials exhibited the formation of a 45° shear band. Such shear localization is a commonly observed failure mode in lattice metamaterials composed of brittle materials [41], as the maximum shear stress under uniaxial compression is typically oriented at 45°. In the present case, however, the shear band formation in DP‐5 arises from architectural design rather than material brittleness, providing a new strategy for engineering metamaterials with tailored failure path or enhanced damage tolerance. Collectively, these observations demonstrate that the heterogeneous arrangement of unit cells along the load path induces localized deformation, and the programmable failure behavior can be deliberately achieved by encoding the spatial distribution of soft and hard elements.

The spatially encoding of the soft and hard elements further enabled the highly accurate and controllable local deformations in a sequential manner, as seen in Figure 3b. The stress distribution within the DP metamaterials was classified into the inactivated and activated regions in terms of a thresholding criterion (5 MPa). The stress values above or below this threshold were considered as ON and OFF states, respectively. Initially, both the soft and hard elements remained in the OFF state. As the strain increased to 0.1, the soft elements were activated and their state switched to the ON state, while the hard elements remained inactive (OFF). When the strain reached 0.4, the hard elements were subsequently activated to ON state. This spatially and temporally controlled activation sequence directly resulted from the encoded soft–hard architecture. Such a programmable stress‐response behavior could be harnessed to design mechanical logic gates or computational metamaterials, in which stress or deformation serves as an input signal to trigger localized mechanical responses in a prescribed sequence.

### 3.3 Machine Learning Inverse Design

In this section, we present a generalized inverse design framework for programming the nonlinear mechanical properties of the DP metamaterials. While preceding designs relied on manual encoding of soft and hard phases within a 16‐element lattice to demonstrate representative behaviors, they served only a small subset of the vast design space enabled by a 4 × 4 grid. In fact, after accounting for geometric symmetry, a total of 16,576 possible configurations remains, each of which can potentially give rise to a unique stress–strain response. This combinatorial complexity underscores the necessity for a generalized and automated inverse design framework capable of systematically generating DP metamaterials with arbitrarily prescribed nonlinear mechanical behaviors.

In the present framework, each DP metamaterial comprises 16 elements, with each element assigned as either soft or hard. Owing to the high degree of architectural programmability and the resulting diversity of mechanical responses, DP metamaterials offer a promising candidate for achieving tailored mechanical behaviors for a wide range of engineering applications, such as quasi‐zero stiffness for vibration isolation or multi‐step plateau responses for energy absorption. To enable rapid design of metamaterials with target mechanical responses, a deep neural network (DNN) was developed to learn the relationship between spatial encoding patterns and the corresponding nonlinear strain‐stress curves. Once trained, the DNN model enables efficient inverse design by identifying optimal coding patterns that reproduce a target mechanical response.

**FIGURE 4:**
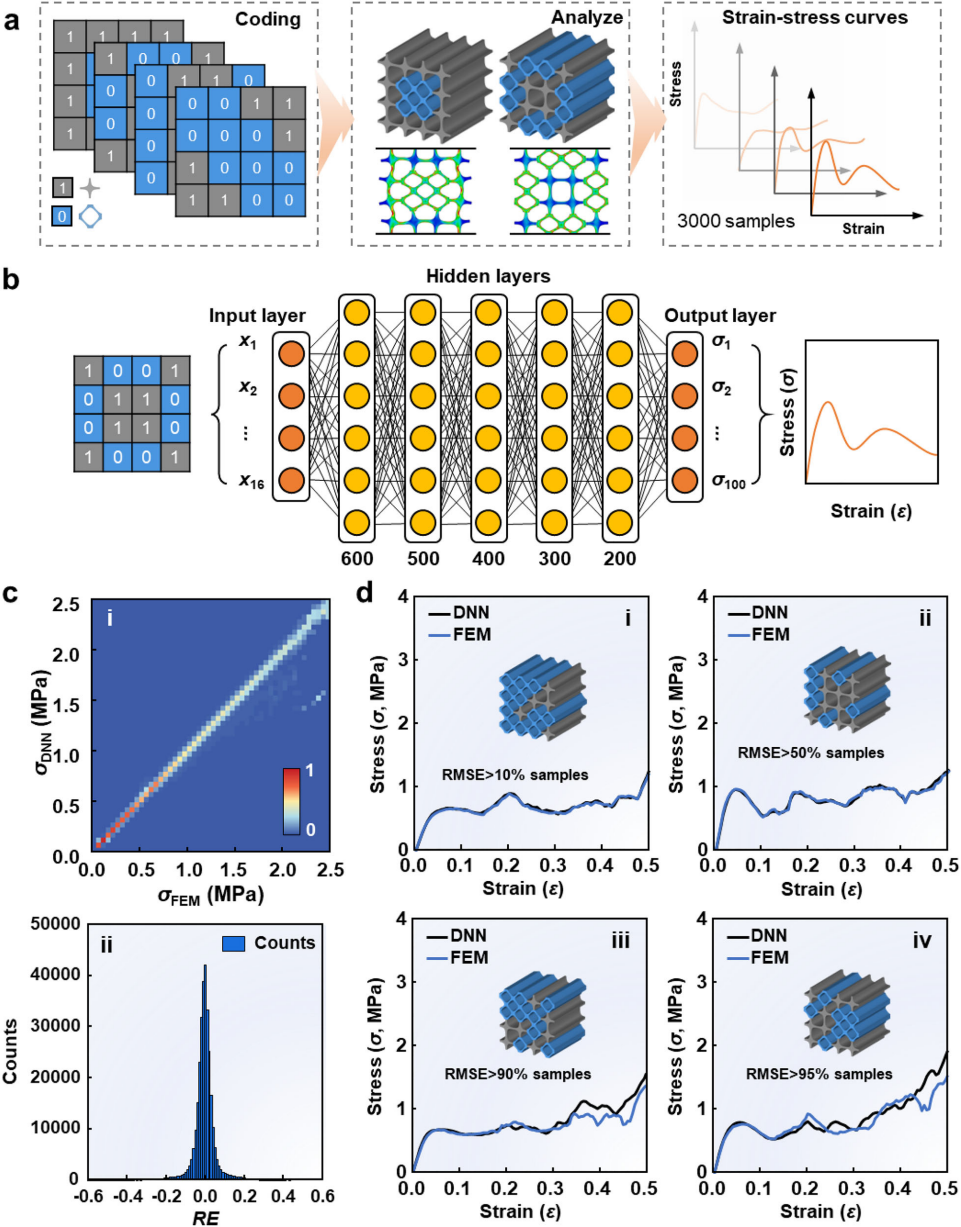
Machine‐learning‐accelerated prediction of mechanical responses for DP metamaterials. (a) Dataset generation for machine leaning, comprising spatial coding patterns and corresponding stress–strain responses. (b) Architecture of the DNN model. (c) Prediction accuracy of the trained DNN model in stress values: (i) comparison of stress values between predicted by FEM‐predicted and DNN, where the color indicates the proportion of data points. (ii) distribution of relative errors in predicted stress values. (d) Prediction accuracy of the trained DNN model for full strain‐stress curves, shown as root mean square error (RMSE) distributions (i) 10%, (ii) 50%, (iii) 90%, and (iv) 95% of the samples.

The dataset comprised the coding patterns and their corresponding strain‐stress curves for 3000 randomly generated DP metamaterial configurations. The metamaterial geometries were generated using a Python code, with the spatial distribution of soft and hard unit cells represented by binary data 0 and 1, respectively. The mechanical responses of these configurations were obtained through FEM, and the corresponding strain‐stress curves as plotted on Figure 4a. The details of the DNN architecture are illustrated in Figure 4b. The input layer contained 16 neurons corresponding to the spatial arrangement of soft and hard elements. The output layer consisted of 100 neurons representing the stress values at 100 strain points. The network included five fully connected hidden layers with rectified linear unit (ReLU) activation functions. The hidden layer parameters were manually chosen to provide sufficient representational capability for capturing the nonlinear relationship between the soft‐hard coding patterns and the corresponding mechanical responses. The dataset was divided into training and validation subsets of 2,100 and 900 samples, respectively; and the model was trained using the adaptive moment estimation (Adam) optimizer.

To evaluate the accuracy and robustness of the trained DNN model, the relative error (*RE*) and root mean square error (*RMSE*) were employed to quantify the deviation between the DNN predictions and FEM simulation results. The equations of *RE* and *RMSE* are defined by:

(2) $$RE=\frac{\sigma_{\mathrm{DNN}}-\sigma_{\mathrm{FEM}}}{\sigma_{\mathrm{FEM}}}\;$$

(3) $$RMSE=\sqrt{\frac{\sum_{i=1}^{N}{\left(\sigma_{i,\mathrm{DNN}}-\sigma_{i,\mathrm{FEM}}\right)}^{2}}{N}}\;$$

where σ_DNN_ and σ_FEM_ denote the stress values predicted by DNN and obtained FEM simulations, respectively. *N* is the number of data points per strain‐stress curve (*N* = 100). Figure 4c compares the 300000 (3000 samples × 100 data points each) stress values from both the training and validation datasets predicted by the DNN against the corresponding FEM results. The majority of the datapoints lie close to the identity line (*y*  =  *x*), with approximately 92.8% of *RE* values falling within ±10%. The results indicate that the trained DNN model can accurately predict the stress values across a wide range of configurations.

In addition to pointwise stress prediction, the predictive performance of the trained DNN model for full strain‐stress curves was assessed, as shown in Figure 4d. The *RMSE* of each predicted strain‐stress curve was calculated. The representative DP metamaterial configurations corresponding to the 10% (case i), 50% (case ii), 75% (case iii), and 95% (case iv) of *RMSE* within the dataset were selected for comparison. Across all these cases, the strain‐stress curves predicted by DNN and FEM were in good agreement, and the hardening and softening features of strain‐stress at large deformations were successfully predicted by the DNN model. These results demonstrate that the trained DNN model can provide an accurate and reliable prediction of strain‐stress responses for given designs of the DP metamaterials. Noteworthy, the developed DNN model constitutes a fully data‐driven framework for predicting the none‐linear mechanical responses of DP metamaterials. Once trained, the DNN model no longer relied on the complex solid mechanics theory or computationally expensive numerical simulations. Therefore, the machine‐learning‐based approach can significantly accelerate the prediction of nonlinear mechanical responses.

**FIGURE 5:**
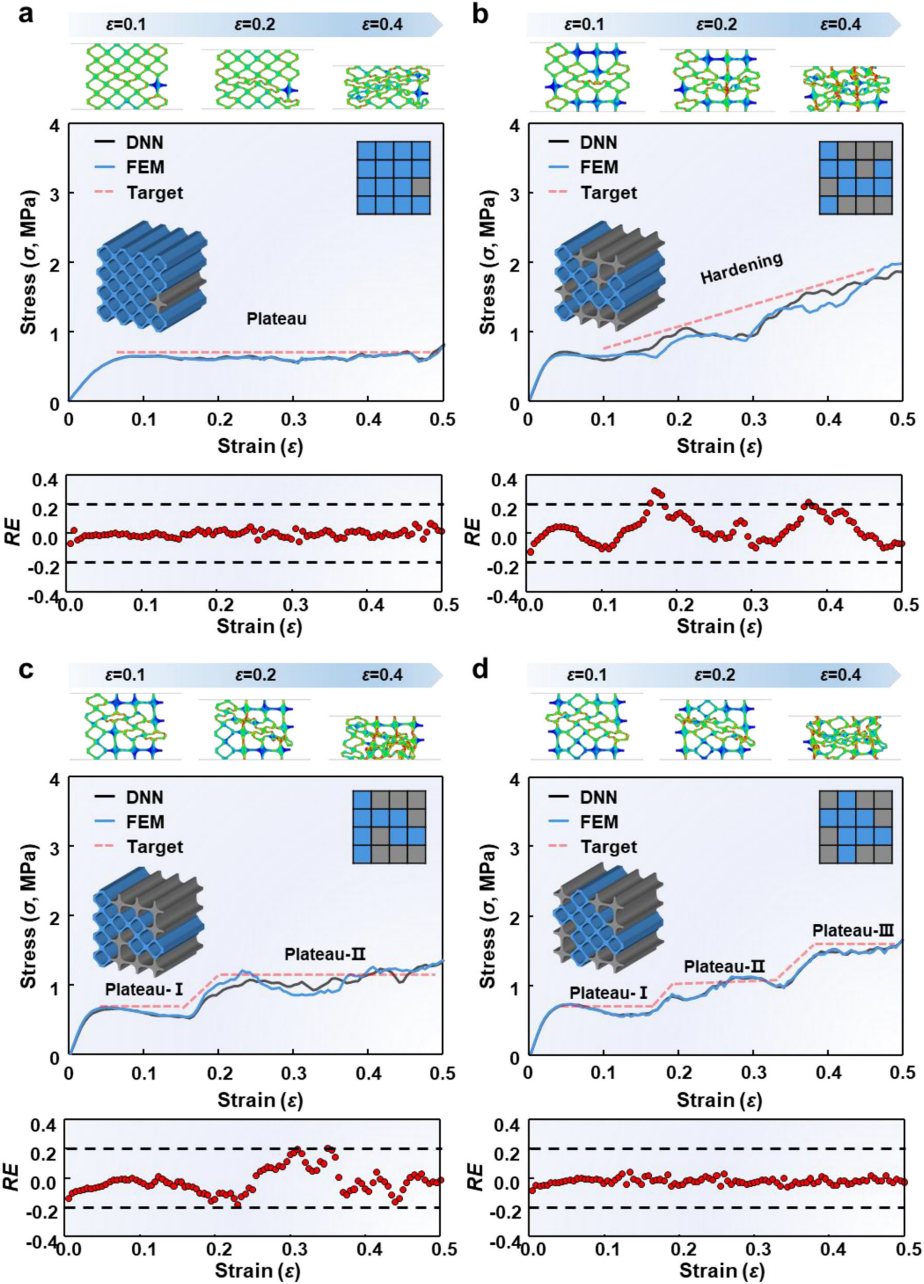
Inverse design of DP metamaterials based on the target compressive responses. Comparison of strain‐stress curves predicted by DNN and FEM for the DP metamaterials exhibiting (a) single plateau, (b) stress hardening, (c) two‐step plateau, and (d) three‐step plateau behavior.

Furthermore, to demonstrate the potential of the trained DNN model for designing DP metamaterials with programmable mechanical responses, four representative target mechanical behaviors were selected, including single plateau, stress hardening, two‐step plateau, and three‐step plateau responses, as illustrated in Figure 5. While a single long and flat plateau is often desirable for maximizing energy absorption efficiency, multiple plateau regions can provide additional functionality through staged energy management. Such behavior is particularly advantageous in automotive crashworthiness applications, where different impact scenarios may require different levels of protection. The mathematical formulations of these target mechanical responses are provided in Section S4 of the Supporting Information. The strain‐stress curves of DP metamaterials with all possible coding patterns were predicted using the trained DNN model, and a comprehensive databank containing all possible coding patterns and their corresponding strain‐stress responses was constructed. The optimal code pattern for each target response was then identified from the databank. Evidently, the trained DNN model can successfully identify feasible coding patterns for all targeted mechanical responses, including the highly complex strain‐stress curves characterized by multiple plateaus. To further validate the inverse design results, finite element simulations were conducted for the inversely‐designed DP metamaterials. The FEM results exhibited good agreement with the DNN predictions, with *RE* values predominantly within ±20%.

Interestingly, the DP metamaterials shown in Figure 5b,c are not included in the original training or validation datasets. The results indicate that the DNN model successfully learned the underlying relationship between the soft‐hard distribution and the corresponding stress‐strain response, rather than simply memorizing the training samples. Overall, this machine‐learning framework provides a powerful and generalizable platform for the inverse design of programmable mechanical metamaterials, enabling rapid discovery of novel metamaterial architectures with targeted mechanical functionalities.

### 3.4 Further Discussion

Inspired by the unique heterogeneous soft‐hard structures observed in the nacre, this study proposed a novel class of DP metamaterials composed of spatially encoded soft and hard elements. Unlike conventional DP metamaterials, which typically rely on multi‐material fabrication techniques [42, 43] or external stimuli to alter material properties [44, 45, 46, 47], the proposed approach achieves tunable mechanical responses through geometry‐based mechanical encoding. This strategy eliminates the need for complex material‐level manipulation and significantly improves compatibility with a broad range of AM technologies.

**FIGURE 6:**
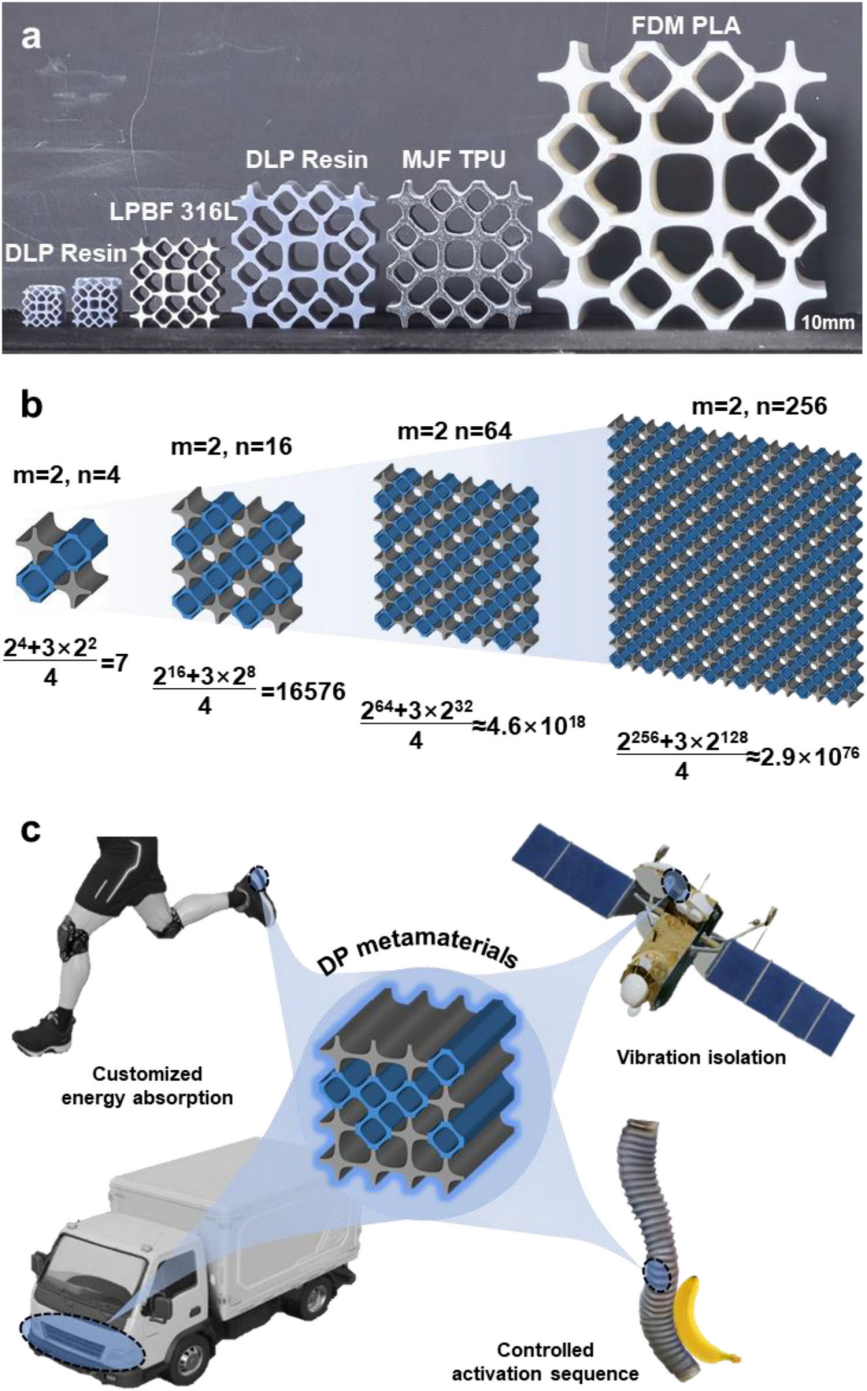
Different types of DP metamaterials. (a) Multi‐scale DP metamaterials fabricated using various AM technologies and materials. From left to right, the fabrication technologies are DLP, DLP, laser powder bed fusion (LPBF), multi jet fusion (MJF), and fused deposition modeling (FDM). The corresponding materials from left to right are resin, resin, 316 stain steel, resin, thermoplastic polyurethanes (TPU), and polylactic acid (PLA). (b) Exponential scaling of coding patterns with number of elements and phases. (c) Representative applications of DP metamaterials.

In the proposed DP metamaterials, the soft and hard phases are realized by assigning distinct topologies of lattice unit cells, enabling fabrication across diverse AM platforms, including fused deposition modeling (FDM, PLA), digital light processing (DLP, resin), laser powder bed fusion (LPBF, 316L stainless steel), and multi‐jet fusion (MJF, TPU), as illustrated in Figure 6a. From a geometric perspective, these architected metamaterials are scalable from the macro‐ to the micro‐scales, subject only to the resolution of the chosen AM process.

Depending on the constituent material and AM platform selected, these DP metamaterials can be tailored for a wide range of application scenarios. For example, DLP‐printed resin supports high‐resolution fabrication of intricate architectures, making it suitable for fine‐scale soft actuators, robotic components, and micromechanical devices. LPBF‐fabricated 316L stainless steel variants offer high strength and high durability, allowing their deployment in load‐bearing structures and energy‐absorbing components for demanding environments such as automotive and aerospace applications. Meanwhile, MJF‐printed TPU offers excellent elasticity and damping characteristics, rendering it ideal for shock‐absorbing footwear, wearable supports, and flexible protective enclosures. Collectively, these diverse examples highlight the broad applicability and material‐agnostic nature of the proposed geometric design strategy across different material platforms and functional requirements.

In contrast to SP metamaterials, whose mechanical responses are fixed and primarily governed by their unit cell topology, the mechanical behaviors of DP metamaterials are influenced not only by the intrinsic properties of the constituent phases but also by their topological arrangement. This introduces a combinatorial design space with substantial programmability. Even within the simplified configuration investigated here (a 4 × 4 array with two phases), the total number of distinct coding patterns reaches 16,576. As illustrated in Figure 6b, for the DP metamaterials composed of *n* elements and *m* phases, the total number of possible coding patterns is given by (*m^n^* + 3*m* ^*n*/2^)/4, which corresponds to an equal number of potential mechanical responses. This result highlights the exponential growth of design possibilities and programmable mechanical behaviors. The details of the total possibilities of coding patterns for DP metamaterials are provided in the Supporting Information, Section S5.

It should be noted that the present study focuses on the 2D TPMS‐based architectures. The DP concept can be readily extended to other architected metamaterials, including conventional lattice structures and honeycomb structures. By selectively integrating soft and hard unit cells within these metamaterials, tailored deformation modes, improved energy absorption, and enhanced mechanical programmability can be achieved. Furthermore, the strategy can be naturally extended to 3D architectures. Such an extension would further broaden the design space and programming potential of DP metamaterials.

Although such rich coding patterns significantly enhance customization potential for engineering applications, it also poses challenges for performance prediction and optimization. Classical rule‐of‐mixture approaches used in composite theory fail to capture the nonlinear, large‐deformation behaviors of architected metamaterials, particularly under energy‐absorbing conditions [48]. As observed, the DP metamaterials with the same soft‐to‐hard phases ratios can exhibit markedly different mechanical responses due to complex phase interactions and stress redistribution mechanisms. These phenomena are not captured by conventional homogenization methods.

Notably, the proposed ML framework based on a DNN model enables rapid and accurate prediction of the nonlinear mechanical responses of DP metamaterials directly from their binary encoding patterns. The prediction accuracy and robustness of the inverse design framework would be further improved by increasing the number of training samples. Additionally, the proposed DNN‐based framework is not limited to the current 4 × 4 array and can be readily extended to more complex DP metamaterials. Once trained, the DNN model provides near‐instantaneous predictions, bypassing the need for computationally intensive nonlinear finite element simulations or analytical modeling. The generation of the 3000‐sample dataset required approximately 25 h, whereas simulating all 16576 configurations would require approximately 138 h. In contrast, DNN training required only approximately 10 min, and the prediction of 16576 configurations took less than 1 s. This approach significantly reduces the design iteration time from several days to a few seconds, which substantially improves the efficiency for engineering applications that require tailored mechanical responses, such as energy absorbers, vibration isolation systems, soft robots, and mechanical computing devices (Figure 6c). For example, energy‐absorbing applications often require multi‐stage mechanical responses to achieve progressive energy dissipation and mitigate catastrophic failure [49, 50]. The vibration isolation systems benefit from extended and flat stress plateaus that emulate quasi‐zero stiffness behavior [51, 52]. Soft robotics and mechanical logic devices further demand a programmable local deformation or sequential activation pattern [53, 54]. As demonstrated in Figure 5, the proposed ML‐assisted inverse design framework can rapidly and reliably generate DP metamaterials that match a wide range of complex target mechanical responses. Noteworthy, this study adopted a DNN model to establish the inverse design framework. Future works will investigate more advanced machine learning models, such as variational autoencoders and generative adversarial networks. These approaches may provide enhanced design diversity and improved optimization capabilities, particularly for more complex metamaterials.

## 4 Conclusion

Inspired by the unique soft‐hard heterogenous architecture observed in natural nacre, this study proposed a novel class of DP metamaterials composed of spatially encoded soft and hard elements. The soft and hard characteristics were realized by using the bending‐ and stretching‐dominated lattice topologies, respectively. Five representative DP metamaterial designs were created by deliberately varying the spatial coding patterns and fabricated using DLP printing. Both the experimental test and numerical simulation results demonstrated that the programmable mechanical responses and failure processes can be achieved by geometric‐based mechanical encoding, governed by phase interactions and internal stress redistribution. Notably, the engineered sequenced failure mechanisms and coupling enhancement effects led to significant improvement in SEA, with certain DP metamaterials outperforming both purely soft and purely hard counterparts. To efficiently and accurately generate DP metamaterials that match a wide variety of complex, nonlinear target responses, a DNN model was developed to learn the relationship between spatial encoding and their corresponding nonlinear mechanical responses. This machine learning‐assisted inverse design framework enables rapid exploration of the vast DP metamaterial design space without reliance on complex solid mechanics theories, substantially accelerating the design process. Overall, this work establishes a general and scalable strategy for programming nonlinear mechanical behavior and failure pathways in architected metamaterials, offering significant potential for engineering applications that demand tailored mechanical responses, including energy absorption, vibration isolation, soft robotics, and mechanical computing devices.

## 5 Experimental Section

### 5.1 Fabrication and Material

The metamaterials were fabricated using a commercial digital light processing (DLP) 3D printer (Anycubic M7, China). A photosensitive resin supplied by the same manufacturer was used throughout this study. All the metamaterial samples had the overall dimensions of 32 mm × 32 mm × 32 mm, consisting of a unit cell with a characteristic size of 8 mm. To ensure a fair comparison between different designs, all the samples were designed with a consistent volume fraction of 0.3.

The DLP printing parameters were set as follows: light intensity of 5.5mw/cm^−2^, exposure time of 2 s per layer, layer thickness of 50 µm. After printing, the samples were rinsed in isopropyl alcohol for 10 min to remove any uncured resin, followed by post‐curing in a UV curing chamber for 1 h. The representative fabricated SP and DP metamaterial samples are shown in Figure S2b,c.

### 5.2 Experimental Evaluation

For each design, two identical specimens were fabricated and tested under uniaxial compression. Compression tests were conducted using a 100kN universal materials test machine to characterize the nonlinear mechanical responses of the metamaterials. The crosshead moved downward at a strain ratio of 0.001s^−1^, and the deformation processes were continuously recorded using a digital camera. The stress‐strain (*σ*‐*ε*) curves of metamaterials were calculated from the force (*F*) and displacement (*S*) data using σ  =  *F*/*A* and ε  =  *S*/*H*, where *A* and *H* are the initial cross‐sectional area and height of the metamaterials, respectively.

### 5.3 Numerical Simulation

The nonlinear mechanical responses of metamaterials were simulated using Abaqus/Explicit. The geometric model of each metamaterial was generated using a Python script and discretized using the CPS4R elements. Two rigid plates, meshed with R2D2 elements, were placed at the top and bottom surfaces of the metamaterials to simulate the compressive boundary conditions (Figure S3a). During the simulation, the top rigid plate was displaced downward by 16mm, while the bottom plate was fixed.

The constitutive mechanical properties of the base resin were obtained from the in‐house uniaxial tensile tests (Figure S3b) and used as input for the simulations. The simulated stress‐strain curves showed good agreement with the experimental results, confirming the validity and the reliability of the simulation model (Figure S4).

## Author Contributions

**Miao Zhao**: conceptualization, investigation, methodology, validation, software, Writing – original draft. **Zhi Zeng**: methodology, supervision, writing – original draft, validation. **Xuhui Zhang**: methodology, formal analysis, visualization, investigation. **Bei Peng**: methodology, writing – review and editing. **Na Qiu**: investigation, methodology, validation, writing – original draft. **Qing Li**: methodology, writing – review and editing, supervision. **Xinwei Li**: conceptualization, supervision, investigation, writing – review and editing.

## Conflicts of Interest

The authors declare no conflicts of interest.

## Supporting information




**Supporting File**: smll74687‐sup‐0001‐SuppMat.docx.

## Data Availability

The data that support the findings of this study are available on request from the corresponding author.
