# Supplementary material for: Geometry‐Encoded Programmable Mechanics in Single‐Material Dual‐Phase Metamaterials
Source: Small. 2026 Jul 24;22(50):e74687. doi: 10.1002/smll.74687 (PMC13548926; doi:10.1002/smll.74687)
Supplement: Supplementary file 1 [file SMLL-22-e74687-s001.docx]

**Supporting Information**

**Geometry-Encoded Programmable Mechanics in Single-Material Dual-Phase Metamaterials**

Miao Zhao^1^, Na Qiu^2^, Xuhui Zhang^1^, Bei Peng^1^, Zhi Zeng^1, *^, Xinwei Li^3, *^, Qing Li^2, *^

1. School of Mechanical and Electrical Engineering, University of Electronic Science and Technology of China, Sichuan 611731, China

2. School of Aerospace, Mechanical and Mechatronic Engineering, The University of Sydney, Sydney, NSW 2006, Australia

3. Newcastle University in Singapore, Faculty of Science, Agriculture, and Engineering, Newcastle University, Newcastle upon Tyne NE1 7RU, UK

*Corresponding authors.

E-mail address: zhizeng@uestc.edu.cn (Zhi Zeng), xinwei.li@newcastle.ac.uk (Xinwei Li), qing.li@sydney.edu.au (Qing Li)

**Supporting Information Text**

S1. Design of metamaterials

S2. Fabrication

S3. Finite element method

S4. Inverse design

S5. Possibilities of coding patterns

# S1. Design of metamaterials

In this study, the surfaces of the lattice metamaterials were represented by the triply periodic minimal surfaces (TPMSs). The geometry of the metamaterials can be controlled by changing the parameters of the TPMS formula. To generate the soft-hard double-phase (DP) metamaterials, the strut‑based primitive lattices act as the “hard” elements, and the shell-based ones serve as the “soft” elements. The TPMS formula of the hard and soft lattices are:

$$f_{\mathrm{hard}}\left( x,y \right)=\cos\left( w_{x}x \right)+\cos\left( w_{y}y \right)+\left( t+10 \right)\left[ \cos\left( w_{x}x \right)\cos\left( w_{y}y \right)+\cos\left( w_{x}x \right)+\cos\left( w_{y}y \right) \right]+t$$

(1)

$$f_{\mathrm{soft}}\left( x,y \right)=\left[ \cos\left( w_{x}x \right)+\cos\left( w_{y}y \right)+t \right]^{2}$$

(2)

where, $w_{i}=2\pi/L_{i} (i=x,y)$ controls the unit cell size, and $L_{i}$ is the unit cell length in the *i* direction. *t* controls the offset position of surfaces, which determines the volume fraction of lattice metamaterials. Then, the solid and void regions of lattice metamaterials are defined by $f\left( x,y \right)\leq0$ and $f\left( x,y \right)>0$, respectively. Figure S1a demonstrates the geometry of soft and hard elements with different volume fractions. In this study, the unit cell size was set to 8×8 mm, and the $L_{i}$=8 mm. The hard and soft elements had the same volume fraction of 0.3, which realized by setting $t_{\mathrm{hard}}$=73.72 and $t_{\mathrm{soft}}$=0.37, respectively.

**Figure S1** The design method for soft-hard metamaterials using mathematical formula. (a) The geometry of soft and hard elements with different volume fractions generated by triply periodic minimal surfaces. (b) The effects of the transition factor on the transition function. (c) The effects of the transition factor on the transition morphologies.

The soft-hard DP metamaterials contain two types of elements with different geometries. To continuously connect the two morphologies, a transition function was introduced to combine different TPMS formula and generate one continuous function. Thus, the mathematical formula of soft-hard DP metamaterials can be expressed as^[1]^:

$$f_{\mathrm{DP}}(x,y)=\sum_{i=1}^{n} \frac{1}{e^{\alpha\left[ {(x-x_{i})}^{2}+{(y-y_{i})}^{2} \right]}}f_{i}\left( x,y \right)$$

(3)

where, $f_{i}\left( x,y \right)$ is the *i*-th element, and $\alpha$ is the transition factor. ${(x}_{i},y_{i})$ is the control point of the *i*-th element. Figures S1b and c illustrate the effects of the $\alpha$ on the morphologies of the transition regions. When the $\alpha$ increased, the value of transition function rapidly decreased from 1 to 0, resulting in a step-wise transition between different morphologies. However, for the small $\alpha$, the disconnect regions were found due to the slowly changed in morphologies. In this study, the $\alpha$ was manually set to 20 to realize a step-wise transition between soft and hard elements. The overall size of the soft-hard DP metamaterials was 32×32×32 mm, which contains 4×4 unit cells along the *x* and *y* directions.

# S2. Fabrication

The DP metamaterials were printed by digital light processing (DLP) as illustrated in Figure S2a. The fabricated hard, soft and DP samples are shown in Figure S2b and c.

**Figure S2** The fabricated samples via DLP. (a) Diagram of the DLP process. (b) The fabricated hard and soft single-phase samples. (c) The fabricated hard-soft DP samples.

# S3. Finite element method

The large deformation behaviors of metamaterials under uniaxial compression were simulated by the finite element method (FEM). The Abaqus/Explicit procedure was used to solve the quasi-static compression process of metamaterials. Figure S3a shows the FEM models of metamaterials. The material properties were obtained from the tensile test, as shown in Figure S3b. The elastic modulus was 696.5±12 MPa, and the yield strength was 11.38±0.1 MPa. The input plastic strain and stress data were obtained from the true strain-stress curve. The general contact with 0.2 friction coefficient was applied for the whole model.

**Figure S3** FEM models of lattice metamaterials. (a) Geometries and boundary conditions used in FEM. (b) True strain-stress curves of the tough resin.

Figure S4 demonstrates the simulated strain-stress curves of hard and soft metamaterials, and the results are compared to the experimental ones. It can be observed that the FEM successfully predicted the none-linear mechanical behaviors of lattice metamaterials.

**Figure S4** Comparison of experimental and simulation results of (a) hard and (b) soft metamaterials

# S4. Inverse design

To demonstrate the potential application of the trained DNN model in designing DP metamaterials with programmable mechanical response, four typical target mechanical responses were selected, including single plateau, stress hardening, two-step plateau, and three-step plateau responses, as demonstrated in Figure S5.

**Figure S5** Four typical target mechanical responses, including (a) single plateau, (b) stress hardening, (c) two-step plateau, and (d) three-step plateau responses

## S4.1. Single plateau

For the single plateau response, the DP metamaterials should have a lower deviation in stress values in the plateau stage. The deviation can be calculated by:

$$D= \sum_{i=10}^{100} \left( \frac{\sigma_{i}-\bar{\sigma}}{\bar{\sigma}} \right)^{2}$$

(4)

where, $\sigma_{i}$ is the stress value in the strain-stress curve, and $\bar{\sigma}$ is the average stress, which can be calculated by:

$$\bar{\sigma}=\frac{\sum_{i=10}^{100} \sigma_{i}}{90}$$

(5)

The DP metamaterials with single plateau response were selected by the curve which had the lowest $D_{S}$.

## S4.2. Stress hardening

For the stress hardening response, the stress values should continuously increase in the plateau stage. The strain-stress data of each curve was fitted using a linear function, and their slop and coefficient of determination ($R^{2}$) were obtained. The DP metamaterials with stress hardening response were selected by the fitting result which had the highest slop and $R^{2}>0.9$.

## S4.3. Two-step plateau

For the two-step plateau response, the DP metamaterials should have two plateaus in the plateau stage, and the second plateau stress should be higher than the first one. Therefore, the relationship between two plateau stress values is:

$$\frac{\bar{\sigma_{2}}}{\bar{\sigma_{1}}}\geq1.6$$

(6)

where, $\bar{\sigma_{1}}$ and $\bar{\sigma_{\mathbf{2}}}$ are the average stress values in each plateau, which can be calculated by:

$$\bar{\sigma_{1}}=\frac{\sum_{i=10}^{45} \sigma_{i}}{35}$$

(7)

$$\bar{\sigma_{2}}=\frac{\sum_{i=45}^{100} \sigma_{i}}{55}$$

(8)

Additionally, the stress values in each plateau should have the lowest deviation, which can be calculated by:

$$D= \sum_{i=10}^{45} \left( \frac{\sigma_{i}-\bar{\sigma_{1}}}{\bar{\sigma_{1}}} \right)^{2}+\sum_{i=45}^{100} \left( \frac{\sigma_{i}-\bar{\sigma_{2}}}{\bar{\sigma_{2}}} \right)^{2}$$

(9)

## S4.4. Three-step plateau

For the three-step plateau response, the DP metamaterials should have three plateaus in the plateau stage. Therefore, the three plateau stress values should meet the requirements:

$$\left\{ \begin{aligned} \frac{\bar{\sigma_{2}}}{\bar{\sigma_{1}}}\geq1.4 \\ \frac{\bar{\sigma_{3}}}{\bar{\sigma_{2}}}\geq1.4 \end{aligned} \right.$$

(10)

where, $\bar{\sigma_{1}}$, $\bar{\sigma_{\mathbf{2}}}$, and $\bar{\sigma_{\mathbf{3}}}$ are the average stress values in each plateau, which can be calculated by:

$$\bar{\sigma_{1}}=\frac{\sum_{i=10}^{45} \sigma_{i}}{35}$$

(11)

$$\bar{\sigma_{2}}=\frac{\sum_{i=45}^{75} \sigma_{i}}{30}$$

(12)

$$\bar{\sigma_{3}}=\frac{\sum_{i=75}^{100} \sigma_{i}}{25}$$

(13)

Additionally, the stress values in each plateau should have the lowest deviation, which can be calculated by:

$$D= \sum_{i=10}^{45} \left( \frac{\sigma_{i}-\bar{\sigma_{1}}}{\bar{\sigma_{1}}} \right)^{2}+\sum_{i=45}^{75} \left( \frac{\sigma_{i}-\bar{\sigma_{2}}}{\bar{\sigma_{2}}} \right)^{2}+\sum_{i=75}^{100} \left( \frac{\sigma_{i}-\bar{\sigma_{3}}}{\bar{\sigma_{3}}} \right)^{2}$$

(14)

# S5. Possibilities of coding patterns

Consider the DP metamaterial consisting of two types of lattices arranged in $n\times n$ elements. Each element can be filled with either hard or soft lattices, leading to $2^{n\times n}$ possible coding configurations if no symmetries are considered. However, in the compression test applied from top to bottom, the configurations that do not alter the loading direction have the equivalent mechanical responses. The relevant symmetry group consists of the four operations: i) identity transformation, ii) horizontal reflection, iii) vertical reflection, and iv) 180° rotation, as shown in Figure S6.

The number of distinct configurations ($N$) can be computed using Burnside’s lemma:

$$N(n)= \frac{\left( X_{id}+X_{hr}+X_{vr}+X_{180^{\circ}} \right)}{4}$$

(15)

where, $X_{g}$ denotes the number of configurations fixed by the symmetry operation $g$.

**Figure S6** Illustration of horizontal reflection, vertical reflection and 180° rotation of coding patterns.

For the identity transformation, all configurations ($n\times n$) are fixed, and the $X_{id}=2^{n\times n}$. For the horizontal reflection, there are $\left( n\times n \right)/2$ configurations are fixed due to the symmetry across the horizontal midline, and the $X_{hr}=2^{n\times n/2}$. Similarly, for the vertical reflection and 180° rotation, there are $\left( n\times n \right)/2$ configurations are fixed, and the $X_{vr}=2^{n\times n/2}$ and $X_{180^{\circ}}=2^{n\times n/2}$. Thus, the Eq. (15) can be expressed as:

$$N(n)= \frac{\left( 2^{n\times n}+3\times2^{n\times n} \right)}{4}$$

(16)

For the multi-phase metamaterials consisted of consisted of $n$ elements and $m$ phases, the total possibilities of coding patterns can be expressed as:

$$N(n,m)= \frac{\left( m^{n\times n}+3\times m^{n\times n} \right)}{4}$$

(17)

# References

[1]	N. Yang, C. Du, S. Wang, Y. Yang, C. Zhang, *Materials Letters* **2016**, 173, 136.
